# Evaluation of a new ELISA assay for monoclonal free‐light chain detection in patients with cardiac amyloidosis

**DOI:** 10.1002/jha2.516

**Published:** 2022-06-24

**Authors:** Hajer Abroud, Asma Beldi‐Ferchiou, Vincent Audard, François Lemonnier, Fabien Le Bras, Karim Belhadj, Anissa Moktefi, Elsa Poullot, Khalil El Karoui, Jehan Dupuis, Alizée Maarek, Louise Roulin, Marie‐Hélène Delfau‐Larue, Silvia Oghina, Mounira Kharoubi, Mélanie Bézard, Amira Zaroui, Thibaud Damy, Valérie Molinier‐Frenkel

**Affiliations:** ^1^ Département d'Hématologie‐Immunologie AP‐HP, Hopital Henri Mondor Creteil France; ^2^ INSERM IMRB Univ Paris Est Creteil Creteil France; ^3^ French Referral Centre for Cardiac Amyloidosis Cardiogen Network GRC Amyloid Research Institute Henri Mondor Hospital Creteil France; ^4^ Unité Hémopathies Lymphoïdes AP‐HP, Hopital Henri Mondor Creteil France; ^5^ Département de Pathologie AP‐HP, Hopital Henri Mondor Creteil France; ^6^ Service de Néphrologie et Transplantation AP‐HP, Hopital Henri Mondor Creteil France; ^7^ Département de Cardiologie AP‐HP, Hopital Henri Mondor Creteil France; ^8^ INSERM, IMRB, CEPiaA Univ Paris Est Creteil Creteil France

**Keywords:** AL amyloidosis, cardiac amyloidosis, ELISA, free‐light chain, monoclonal gammopathy

## Abstract

The causal protein of amyloid light‐chain (AL) amyloidosis is a monoclonal immunoglobulin free light chain (mFLC), which must be quantified in the serum for patient diagnosis and monitoring. Several manufacturers commercialize immunoassays that quantify total kappa (κ) and lambda (λ) FLC, but results can differ greatly between these tests. Here, we compared a recently developed enzyme‐linked immunosorbent assay (ELISA) (Sebia) with N‐Latex immunonephelometry (Siemens) in 96 patients diagnosed with AL amyloidosis (histologically confirmed) and 48 non‐AL patients sent to our referral center for suspicion of cardiac amyloidosis. ELISA free‐light chain difference (dFLC) were lower than N‐Latex values, and agreement between methods was reduced in the case of involved λ FLC. Diagnosis sensitivity and specificity were >85% with both assays. A receiver operating characteristic analysis indicated that ELISA performances could be improved by using a higher value for the lower limit of the κ/λ ratio. We also assessed Freelite (The Binding Site) in a subgroup of these same AL patients, including 18 cases with normal κ/λ ratio by at least one assay. Only two patients had normal κ/λ ratio with all three assays. Overall, ELISA demonstrated slightly lower sensitivity than N‐Latex but may be an alternative to nephelometry/turbidimetry in certain difficult cases.

## 1 INTRODUCTION

Systemic amyloid light‐chain (AL) amyloidosis is a rare disease caused by a monoclonal gammopathy in which the deposition of misfolded monoclonal immunoglobulin free light chains (mFLC) results in the disruption of tissue structure [1, 2, 3]. Renal and cardiac involvement are the two main clinical manifestations, the latter being closely associated with patient mortality [4]. Therefore, one of the major issues in AL amyloidosis management is to perform early diagnosis before the occurrence of irreversible organ damage [1, 4–6].

AL diagnosis currently relies on Congo red staining of a tissue biopsy followed by FLC κ or λ identification. Electrophoresis (EP) and immunofixation of the serum and urine allow detection of the underlying plasma cell dyscrasia. As mFLC production is generally weak and can be the exclusive manifestation of the monoclonal gammopathy, these analyses are completed by techniques that sensitively quantify total κ and λ FLC in the serum to detect an excess of the involved type (iFLC). The quantification is based on immunodetection of conserved regions of polyclonal κ and λ FLC that are accessible only when these light chains are not linked to immunoglobulin heavy chains [7]. Diagnosis of monoclonality by this method requires both an increased concentration of the iFLC and corresponding imbalance of the κ to λ ratio (κ/λ ratio) in comparison to reference values. The concentration of the mFLC is then approximated by calculating the difference (dFLC) between iFLC and uninvolved FLC (uFLC). Current guidelines recommend using dFLC for monitoring under specific therapy [1, 8, 9].

The quantification of serum FLC is very sensitive and specific but suffers from two main drawbacks. Firstly, in the case of chronic kidney disease (CKD), the half‐lives of total κ and λ FLC increase to a highly variable degree, interfering with mFLC measurement [10]. Secondly, several assays, which rely on different reagents, methods, and devices, are available. As mFLC are unique to each patient, the quantification of an individual mFLC can vary greatly depending on the technique used [11, 12, 13].

At least four manufacturers currently commercialize automated immunoassays: The Binding Site, Siemens, Diazyme, and Sebia [7]. The assays use FLC‐specific polyclonal antibodies from various sources, except for N‐Latex, which uses pairs of monoclonal antibodies from a mouse source. Monoclonal antibodies greatly limit inter‐lot variability [14] but can miss certain polymerized mFLC. Most of the tests measure the presence of the soluble immune complexes by detecting the changes in turbidity through nephelometric or turbidimetric methods. These methods can suffer from high imprecision at low concentrations, nonlinear immunoreactivity on dilutions, and/or the inability to detect antigen excess, leading to underestimation of mFLC concentration [14, 15, 16, 17], all of which can potentially impact the κ/λ ratio. Freelite from The Binding Site was the first available method in 2001 and has been developed as six independent tests adapted to different nephelometry and turbidimetry analyzers. These tests differ in several parameters, such as reaction solution and buffers, antibody dilutions, and starting dilutions for the serum sample. Half of the instruments performing Freelite tests are not programed to detect antigen excess. In contrast to Freelite, there is a single N‐Latex FLC test from Siemens, usable on all three Siemens nephelometers. Although mass spectrometry techniques have recently been developed to overcome the bias of immunodetection, there is no reference method currently available for accurate quantification of FLC in all patients [18, 19]. To overcome some of the analytical problems of nephelometry/turbidimetry, Sebia has developed a sandwich enzyme‐linked immunosorbent assay (ELISA) usable on most ELISA platforms or performed manually. Two‐step sandwich ELISA is a robust and sensitive method that is not affected by antigen excess. The new FLC test presents the advantages of high reproducibility, linearity, and a wide measurement range, which is particularly adapted to measuring mFLC [20].

In this study, we investigated the performance of the recently commercialized FLC ELISA test from Sebia for the diagnosis and quantification of amyloid mFLC in our reference center. One hundred forty‐four patients who had a suspicion of cardiac amyloidosis were tested. Among them, 96 patients displayed definitive diagnosis of AL amyloidosis, and 48 were used as controls. Results were compared with those obtained using the N‐Latex FLC assay routinely used in our laboratory, and with the Freelite assay in some patients. The follow‐up of five AL patients receiving chemotherapy was also compared between N‐Latex and ELISA.

## 2 MATERIAL AND METHODS

### 2.1 Patients

This study was conducted retrospectively from March 2016 to September 2019 on serum samples from patients suspected of having cardiac amyloidosis who were referred to the Referral Center for Cardiac Amyloidoisis in Henri Mondor Hospital (Créteil, France). All patients signed informed consent. Serum and urine samples were obtained at the initial phase of explorations, before definitive diagnosis and treatment. Patients with previous diagnosis of multiple myeloma were excluded from the study. Diagnostic criteria for amyloidosis have been previously described [21]. The AL cohort included patients with biopsy‐proven (histological characterization, immunohistochemistry, and/or proteomic analysis) κ or λ light chain‐type AL amyloidosis. For five of them, a follow‐up study was also performed on serum samples collected after initiation of chemotherapy.

The control cohort included patients with histologically‐confirmed transthyretin or serum amyloid A (AA) amyloidosis, or hypertrophic cardiomyopathy unrelated to amyloidosis. Patients with a monoclonal immunoglobulin detectable by immunofixation electrophoresis (IFE) in the serum or urine and without AL amyloidosis were excluded from the control cohort. Therefore, the presence of a circulating mFLC was a priori restricted to patients from the AL cohort, and a κ/λ ratio out of range in the control cohort could be considered as a false positive result.

### 2.2 EP, immunofixation, and FLC quantification

For each patient, EP, IFE, and all FLC assays were performed on the same serum sample. Serum EP (sEP) was performed on a Capillarys 2 and IFE (serum and urine) on a Hydrasys 2 scan focusing, both from Sebia (Lisses, France). Unconcentrated urine samples were analyzed on Hydragel Urine Profile.

Serum κ and λ FLC concentrations were measured by ELISA (Sebia) on an ELISA ELITE automated system from DAS, and by nephelometry using N‐Latex FLC (Siemens Healthcare) on a BN‐ProSpec from Siemens. Nephelometric analyses using Freelite (The Binding Site) were also performed on BN‐ProSpec in 25 patients with AL amyloidosis for whom a sufficient volume of serum was available. FLC were initially quantified by N‐Latex and/or Freelite on fresh serum (routine testing) that was then stored at −80°C and thawed at the time of further FLC analysis. ELISA was performed blind by Sebia on thawed anonymized serum in six independent runs. Coefficients of variation of polyclonal controls within‐run and between runs did not exceed 5.0% and 8.0% respectively for all three techniques.

The κ/λ ratio and dFLC were calculated for each patient. The dFLC is defined as the difference between iFLC and uFLC and was calculated as│κ‐λ│(absolute value of the difference between κ and λ FLC concentrations). The reference intervals of each test are provided in Table 1.

**TABLE 1:** Reference values for free light chain (FLC) quantification tests provided by the manufacturers

	κ (mg/L)	λ (mg/L)	κ/λ ratio	Renal κ/λ ratio^1^
ELISA	5.15–15.30	8.23–18.10	0.37–1.44	0.46–2.23
N‐Latex	6.7–22.4	8.3–27.0	0.31–1.56	–
Freelite	3.30–19.40	5.71–26.30	0.26–1.65	0.37–3.1

^1^ Adapted renal reference ranges have been established in patients with chronic kidney disease (CKD) for κ/λ ratio measured with Freelite and ELISA, as values increase following renal impairment in the absence of dysglobulinemia [37, 41]. This does not occur with N‐Latex [28]. In the present study, the renal reference range was applied for patients with eGFR <60 ml/min/m^2^ (CKD stage > 2).

### 2.3 Statistical analysis

Statistics were performed as specified in the text and figure legends, using Graphpad Prism8, and using XLSTAT for Passing‐Bablok analyses.

The diagnostic performance of each FLC quantification assay was measured as the capacity to detect the underlying mFLC gammopathy in AL patients (abnormal κ/λ ratio in relation to iFLC) and to report normal κ/λ ratio in control patients. Thus, classification of the patients as true κ or λ positive and false negative were determined in the AL cohort, and true negative and false positive were determined in the control cohort, before calculating sensitivity and specificity [22].

## 3 RESULTS

### 3.1 Study population

Table 2 summarizes the characteristics of all patients. The AL cohort included 96 patients with primary AL amyloidosis of whom 78 had λ iFLC, and 18 had κ iFLC. A monoclonal gammopathy (intact immunoglobulin and/or FLC) could be detected by sEP and/or serum IFE (sIFE) in 84 of the samples. The iFLC was visible on sIFE in 64 (67%), including seven cases with detectable peak on sEP. The control cohort included 48 patients with wild‐type or mutated transthyretin amyloidosis (*n* = 34), AA amyloidosis (*n* = 1), or hypertrophic cardiomyopathy unrelated to an amyloidosis process (*n* = 13). Control patients were older and had less severe renal and cardiac disease.

**TABLE 2:** Demographic clinical characteristics and laboratory data of the patients

		AL κ (*n* = 18)	AL λ (*n* = 78)	Controls (*n* = 48)
Demographic parameters	Age at diagnosis (IQR)	67 (62; 74)	68 (59;76)	78 (71; 83)
	Men (%)	8 (44)	48 (62)	36 (75)
Kidney	Median eGFR (IQR)	51 (33; 69)	66 (47; 81)	62 (45; 72)
	eGFR <60 ml/min/1.73 m^2^	13 (72.2)	34 (43.6)	22 (45.8)
	Dialysis (%)	1 (5.6)	2 (2.5)	0 (0)
	Proteinuria >3 g/24h (%)	2 (11.1)	10 (12.7)	1 (2.1)
Heart	Median cTnT, ng/L (IQR)	100 (53; 236)	93 (57; 138)	43 (23; 78)
	Median NT‐proBNP, ng/L (IQR)	7333 (2118–14543)	6204 (2771–11242)	1912 (661–3257)
	Median strain (IQR)	−9.8 (−13.4; −6.8)	−9.5 (−11.3; −7.6)	−9.6 (−12.0; ‐7.4)
Monoclonal gammopathy	sEP peak (%; median size)	7 (39; 5 g/L)	47 (60; 8 g/L)	–
	Intact monoclonal Ig (%)	7 (39)	42 (54)	–
	mFLC detection by sIFE (%)	13 (72.2)	51 (64.6)	–
Wild‐type TTR amyloidosis (%)		–	–	28 (58)
Mutated TTR amyloidosis (%)		–	–	6 (13)
HCM unrelated to amyloidosis/AA amyloidosis (%)		–	–	14 (29)

*Note*: Data are presented as the median (with interquartile range, IQR) or the number of patients concerned (with percentage of the corresponding cohort). Under monoclonal gammopathy the data presented are: the number of patients with a peak detectable by sEP (with percentage and median value of the peak in g/L), the number of patients with an intact monoclonal immunoglobulin (with percentage), and the number of patients with a monoclonal FLC detectable (with percentage).

Abbreviations: eGFR, estimated glomerular filtration rate by MDRD equation (ml/min/1.73 m^2^); cTnT, cardiac troponin T; NT‐proBNP, N‐terminal fragment of the prohormone brain‐type natriuretic peptide; sEP, serum electrophoresis; sIFE, serum immunofixation electrophoresis; TTR, transthyretin; HCM, hypertrophic cardiomyopathy; IQR, interquartile range.

### 3.2 ELISA values are correlated with, but lower than, N‐Latex values

**FIGURE 1:**
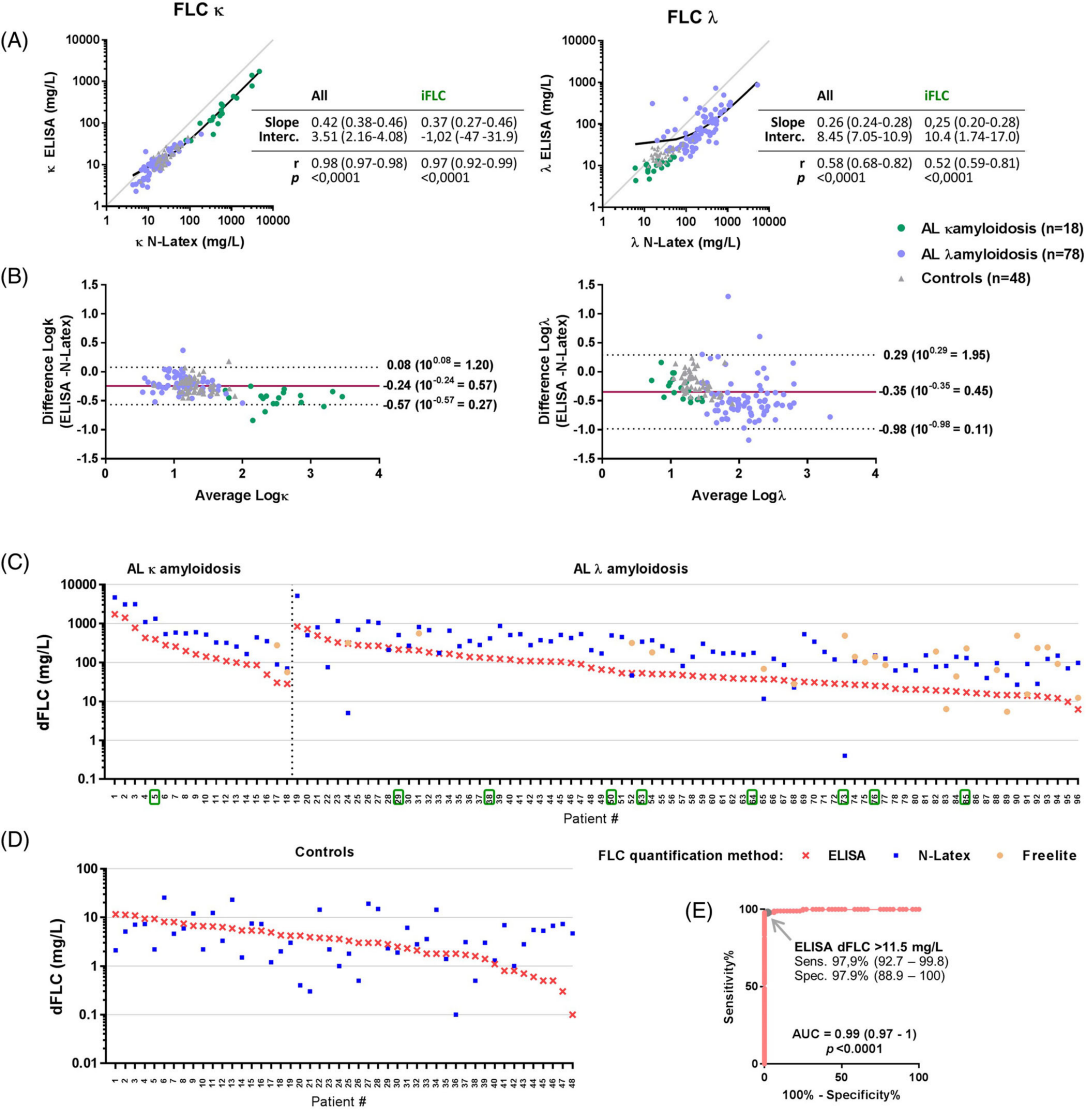
**Raw** free light chain (FLC) **data and dFLC values of the whole cohort**. (**A**) Linear regression analyses of FLC κ (left) and λ (right) values obtained by ELISA and N‐Latex presented on a Log scale. Points represent patients with AL amyloidosis (green, κ AL; light blue, λ AL), and grey triangles represent controls. Grey lines represent perfect agreement. Passing‐Bablok slope coefficient and intercept with 95% CI, Pearson r for linear fitting with 95% CI and *p* value, were calculated for all values and iFLC only. (**B**) Bland‐Altman plots of the agreement between N‐Latex and ELISA values for κ (left) and λ (right) FLC. The x‐axis displays the mean of Log‐transformed FLC values obtained by ELISA and N‐Latex ([LogFLC ELISA + LogFLC N‐Latex]/2); the y‐axis displays the difference (LogFLC ELISA ‐ LogFLC N‐Latex). The horizontal plain line shows the mean of the differences, and the dotted horizontal lines show the 95% CI (with back transformation of the Log values). (**C and D**) The dFLC were calculated for the 96 AL patients (**C**) and the 48 controls (**D**) using κ and λ FLC values measured with ELISA (red x) and N‐Latex (blue dots). Results were also obtained from Freelite quantification in 25 patients with AL amyloidosis (orange dots). The vertical dotted line in A separates patients with κ (*n* = 18) and λ (*n* = 78) AL amyloidosis. Numbers on the x‐axis corresponds to individual patients. The patients were ordered according to dFLC ELISA value (from largest to smallest). Framed numbers refer to patients’ serums displaying a peak on sEP (0.1 to 0.4 g/L, tangential skim) corresponding to the mFLC. (**E**) Receiver operating characteristic (ROC) analysis of ELISA dFLC values from the 144 patients. The area under the curve (AUC), *p* value, and results for a selected cut‐off point with 95% CI are reported

We first measured serum κ and λ FLC by ELISA and N‐Latex and compared results obtained by both methods in the whole cohort (AL and controls). N‐Latex and ELISA were performed on the same aliquot of serum, freshly collected or thawed from −80°C respectively, but this should not have led to significant bias [23]. Results correlated closely, but ELISA values were proportionally lower than N‐Latex values by Passing‐Bablok linear regression analysis (Figure 1A). A Bland‐Altman plot of Log‐transformed values was constructed to allow calculation of the proportional bias (Figure 1B). After back transformation, it was found that the mean difference (ELISA ‒ N‐Latex) represented an average of 0.57‐fold and 0.45‐fold of the mean κ and λ value, respectively. This bias was accentuated for the highest values (iFLC) of AL patients (Figure 1B). Lower linear fitting and a wider range of differences was seen for λ FLC, particularly for the monoclonal ones.

We next calculated dFLC in the AL cohort, as it represents an estimate of the circulating fraction of the amyloidogenic mFLC. Figure 1C displays the results for each of the 96 patients. For 25 of them, values were also obtained using Freelite (see below for description). ELISA dFLC was lower than N‐Latex dFLC in most patients, with a median percent decrease of 63.9% for κ AL and 75.5% for λ AL. Of note, ELISA and N‐Latex dFLC values were around three‐fold higher in patients with κ AL amyloidosis compared to patients with λ AL amyloidosis. Nine patients displayed a detectable peak on sEP corresponding to the mFLC (framed patient numbers on Figure 1C; peak value ∼0.1 g/L in 7/9 cases, ∼0.2 g/L in #50, ∼0.4 g/L in #29). However, high dFLC (> 1000 mg/L; *n* = 9 with N‐Latex, *n* = 2 with ELISA) were associated with mFLC peak in only one case (#5; >1000 with both tests). Thus, assuming high dFLC would naturally correspond to a detectable peak on sEP, N‐Latex may be overestimating the mFLC more frequently than ELISA.

Current guidelines regarding dFLC values usable for monitoring amyloidosis patients who are receiving therapy propose a cut‐off of 50 mg/L based on Freelite performed on a BNII instrument. Using this cut‐off, 92% and 54% of AL patients may have measurable disease using N‐Latex and ELISA, respectively.

For comparison, we also calculated the dFLC in the control cohort, despite it having no clinical significance. All cases displayed very low values (median 3.2 mg/L and 3.5 mg/L, for N‐Latex and ELISA respectively) (Figure 1D), and a receiver operating characteristic (ROC) curve analysis indicated that AL patients were distinguished from controls with high sensitivity and specificity by an ELISA dFLC > 11.5 mg/L (Figure 1E). Thus, patients with ELISA dFLC lower than 50 mg/L, for example, in the range of 25 mg/L, may benefit from ELISA follow‐up.

### 3.3 Sensitivity and specificity of Sebia FLC

**FIGURE 2:**
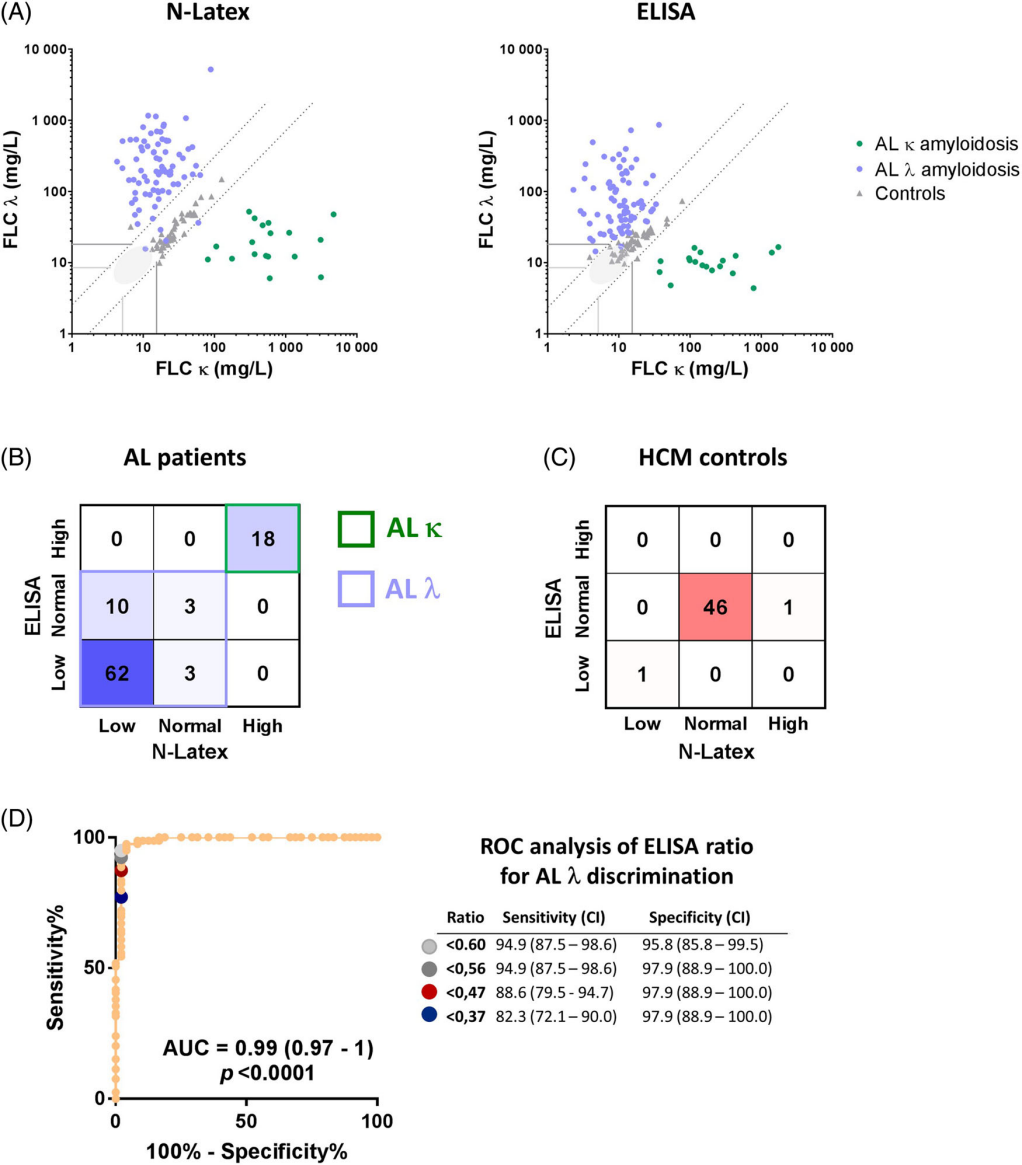
**Concordance analysis and diagnostic performance**. (**A**) Free light chain (FLC) concentrations measured by N‐Latex and ELISA in patients with AL amyloidosis (green, κ AL; light blue, λ AL) and in control patients (grey triangles). The grey lines delimit lower and upper reference values for FLC concentration and κ/λ ratio; points in the light grey area have normal FLC concentrations and κ/λ ratio. (**B and C**) Clinical concordance analysis of the κ/λ ratio between ELISA and N‐Latex within the AL cohort (B) and the control cohort (**C**). In B, green and blue frames delimit results for κ and λ AL amyloidosis, respectively. (**D**) Receiver operating characteristic (ROC) curve analysis of ELISA κ/λ ratio values from λ AL and control patients. The AUC, *p* value, and results for four selected cut‐offs points with 95% CI are reported

We next investigated the concordance of clinical interpretation between ELISA and N‐Latex (Figure 2). The value of Cohen's kappa coefficient (0.84, 95% CI = 0.76–0.92) indicated a very good agreement between both tests. We observed perfect agreement for κ AL (Figure 2A, green dots), with 100% κ/λ ratio above reference range (Figure 2B) and very good agreement for control samples (Figure 2A, gray dots and 2C). The sensitivity of both tests decreased for λ AL, as 16 samples (20% of the λ AL cases) had normal κ/λ ratio, including three samples that were normal by both tests (Figure 2B).

We then calculated the sensitivity and specificity of FLC tests based on ratio results, and the sensitivity of sIFE and uIFE for mFLC detection (Table 3). FLC tests displayed high specificity (>95%). The sensitivity of ELISA was lower than that of N‐Latex. However, a ROC curve analysis indicated that better distinction between λ AL patients and controls (enhanced sensitivity without loss of specificity) could be obtained using a cut‐off of 0.56 as the lower reference value for the κ/λ ratio (Figure 2D). Combining FLC tests with sIFE alone or in combination with uIFE enhanced their sensitivity to >95% (Table 3).

**TABLE 3:** Diagnosis performance

	Sensitivity %	Specificity %
N‐Latex	93.8 (87.0–97.1)	95.8 (86.0–99.3)
ELISA	86.4 (78.2–91.9)	97.9 (89.1–99.9)
sIFE	64.6 (54.6–73.4)	
uIFE	67.7 (57.8–76.2)	
N‐Latex + sIFE	96.9 (91.2–99.2)	
ELISA + sIFE	95.8 (89.8–98.4)	
N‐Latex + sIFE + uIFE	100 (96.2–100)	
ELISA + sIFE + uIFE	95.8 (89.8–98.4)	

*Note*: Percent sensitivity and specificity (with 95% confidence interval) were calculated using κ/λ ratio reference intervals reported in Table 1. Sensitivity was also calculated for the detection of mFLC by immunofixation electrophoresis of the same serum (sIFE) and of a urine sample (uIFE) from the same patient, alone or in combination with FLC quantification. Due to the selection of patients without detectable monoclonal component in the serum or urine by EP and IFE for the control cohort (see material and methods), the specificity could not be calculated for these methods.

### 3.4 Agreement of Sebia ELISA and N‐Latex during monitoring

**FIGURE 3:**
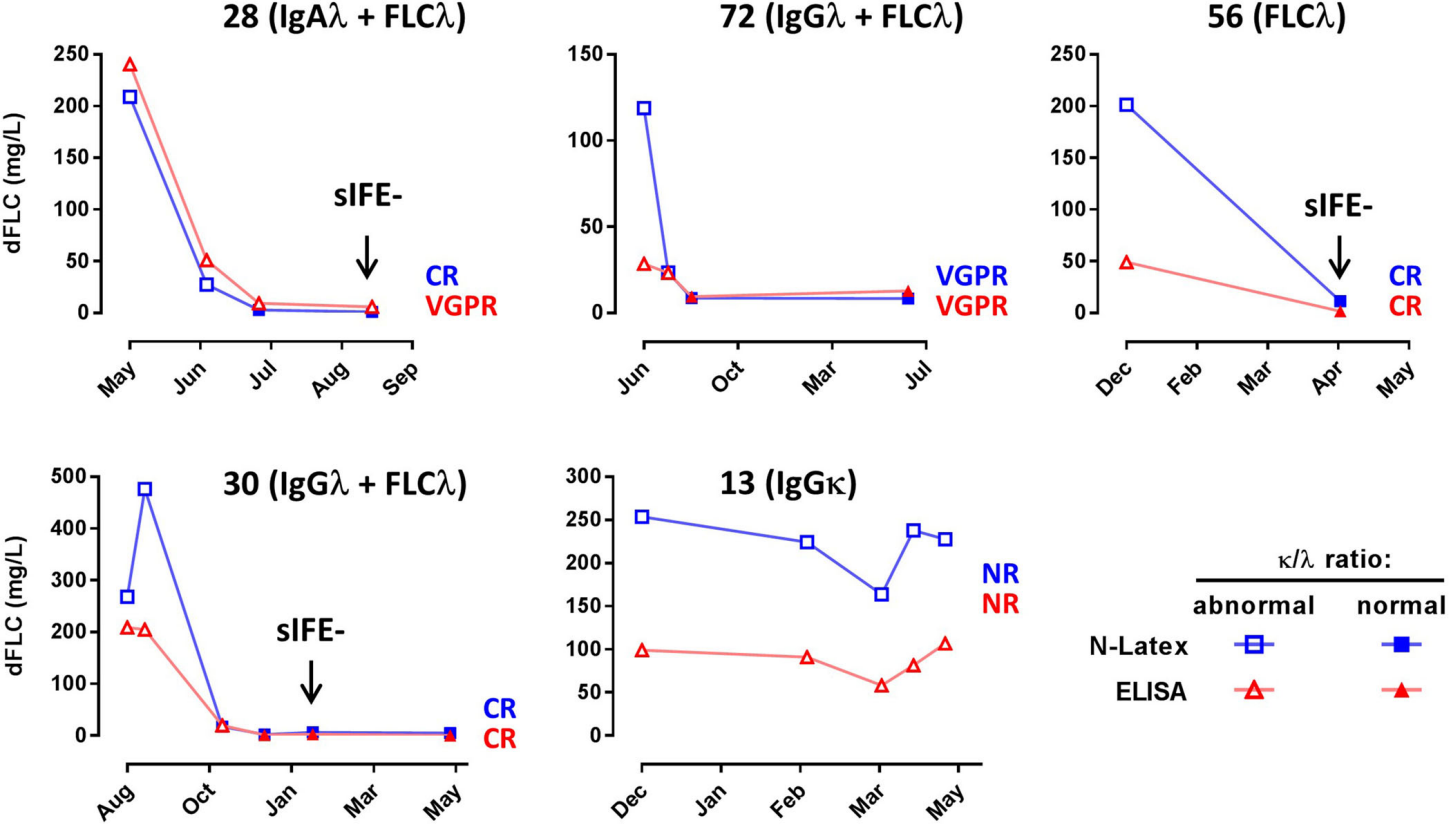
**Coherence of ELISA and N‐Latex results during monitoring of AL amyloidosis patients**. The follow‐up under treatment of five AL amyloidosis patients using dFLC values calculated using N‐Latex and ELISA is shown. The first time point corresponds to diagnosis. Monoclonal components detected on sIFE are indicated for each patient. All patients, except #56, initially had a peak of entire IgG or IgA on serum electrophoresis. Triangles represent ELISA and squares, N‐Latex, with filled and open symbols referring to normal and abnormal κ/λ ratios, respectively. The black arrow indicates the time of first negative sIFE (disappearance of monoclonal component(s) detected at diagnosis). The hematologic response at the last time point is indicated (blue, N‐Latex; red, ELISA). CR, complete remission (negative sIFE and uIFE, normal FLC level and κ/λ ratio); NR, no response; VGPR, very good partial response (dFLC reduction > 50%)

We next compared the kinetics of dFLC values obtained with ELISA and N‐Latex in five patients of our AL cohort (Figure 3). Although dFLC were higher with N‐Latex before treatment in all patients except one, both tests generally showed the same curve shapes, and normalization of ELISA and N‐Latex κ/λ ratio occurred at the same time in two out of the four patients that responded to therapy (patients 72 and 56). Patients 28 and 30 normalized their κ/λ ratio earlier with N‐Latex than with ELISA. These minor discrepancies would not be expected to affect the clinical decisions, as both patients still had detectable monoclonal components by sIFE at the time of N‐Latex FLC normalization.

### 3.5 Comparison of the diagnosis performance of three FLC assays in challenging patients from the AL cohort

**FIGURE 4:**
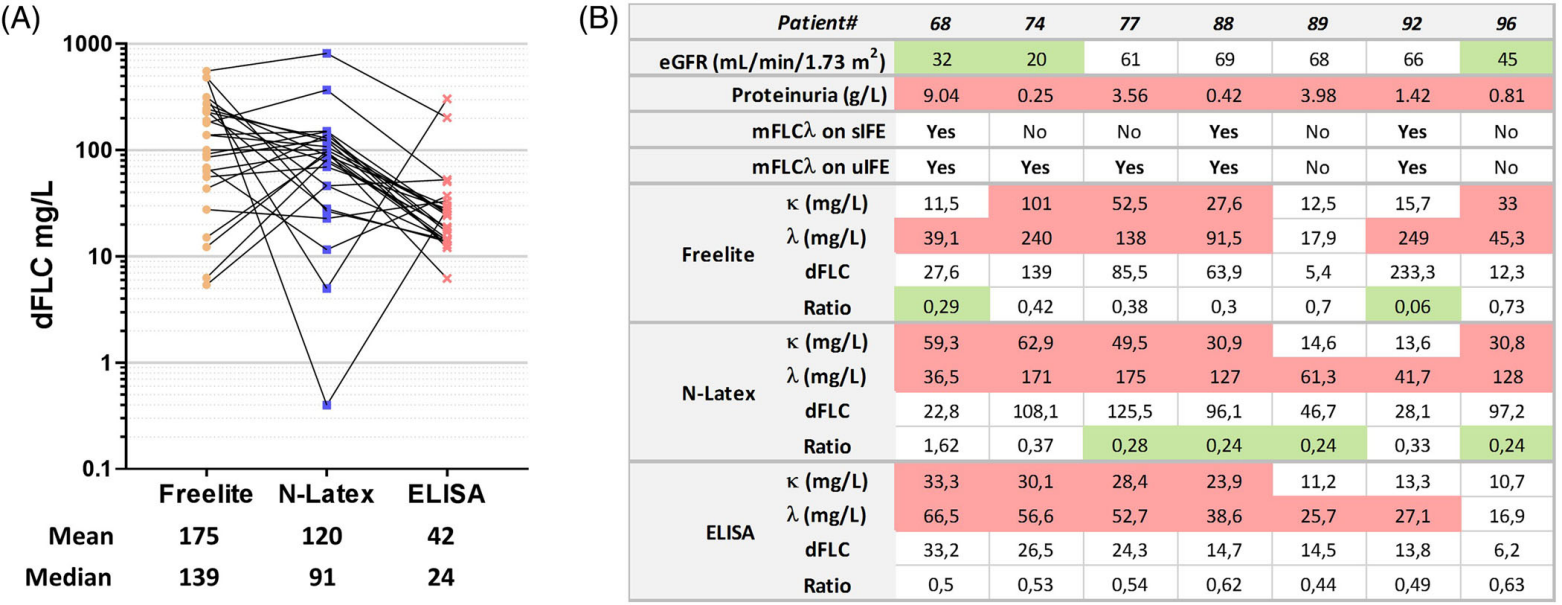
**Comparative analysis using Freelite, N‐Latex, and ELISA in a sub‐cohort of AL patients including difficult cases**. (**A**) The dFLCs were calculated for 25 patient samples analyzed with each FLC test. Yellow circles, blue squares, and red x represent values obtained with Freelite, N‐Latex, and ELISA, respectively. A black line links results from the same patient. (**B**) FLC results, presence of an mFLC on sIFE, proteinuria, and eGFR (MDRD equation) in seven patients—all with λ AL amyloidosis—that were misdiagnosed by more than one FLC test. Green and red background indicate values below and above reference range, respectively

In 25 patients of the AL cohort, the Freelite quantification could also be performed. Samples included those cases with diagnostic difficulties (*n* = 18) (Figure 4). The mean and median values for dFLC at diagnosis greatly differed between the three tests, with Freelite displaying the highest values. Four AL λ cases had normal κ/λ ratio with low dFLC (<50 mg/L) by N‐Latex. For two of them (patients 73 and 92), serum was available for reanalysis using a low dose of a reducing agent (dithiothreitol [DTT] 15 mM, 7 min RT), which can facilitate detection by depolymerizing monoclonal λ FLC. Dithiothreitol (DTT) treatment increased both κ (uFLC) and λ (iFLC) values, the latter in much higher proportions, shifting the ratios to abnormally low values (0.04 and 0.26, respectively), thereby revealing the underlying monoclonal gammopathy. However, DTT treatment cannot be used for patient monitoring, and ELISA may be an alternative method in this case.

Seven of these 25 patients were missed by more than one test, despite biopsy‐proven amyloidosis (Figure 4B). All seven had λ iFLC. None of them were treated by intermittent hemodialysis, but all exhibited proteinuria and decreased eGFR. Three had mFLC on sIFE and five on uIFE. Misdiagnosis could be due to analytical limitations of each test, as in the case of polymerized λ FLC, which can be poorly detected by N‐Latex (patient 92). Alternately, renal impairment can induce retention of both uFLC and iFLC, as occurred in patient 74 (severe renal insufficiency), who had undetected serum amyloid FLC by all methods (normal ratio, no mFLC on sIFE), but their dFLC values may be high enough for monitoring. Major proteinuria may also interfere with quantification by accelerating iFLC elimination, as with patient 68 who had low uFLC (11.5 mg/L) and weak dFLC (27.6 mg/L) with Freelite, despite stage 3b CKD.

These complicated cases illustrate that using more than one FLC quantification method can optimize detection of the amyloid FLC in the serum of a minority of patients with λ AL.

## 4 DISCUSSION

Until recently, AL amyloidosis was considered an incurable disease with poor patient outcome [24]. The detection of the underlying monoclonal gammopathy relies on multiple tests, but FLC quantification is the most sensitive and has become essential to the management of patients [25]. Our study evaluates, for the first time, the performance of a recently commercialized ELISA assay from Sebia in the environment of patients with either definitive diagnosis of AL amyloidosis or non‐AL HCM, all initially addressed for suspicion of cardiac amyloidosis.

Several methods for κ and λ FLC quantification have been developed since the commercialization of the first Freelite test by The Binding Site. Due to analytical differences in the current methodologies, individual patient results, clinical cut‐offs, and reference values are strictly test specific. Both Siemens and Sebia have published normal reference ranges for their own FLC assays [20, 26]. The Binding Site has provided reference values only for quantifications performed on the BNII nephelometer [27] and recommends on its technical datasheet that reference ranges be determined by users of other detection devices. With Freelite and ELISA, specific reference ranges of the κ/λ ratio have been determined for patients with CKD, whereas N‐Latex ratio values are similar in patients with renal failure and healthy controls, despite there being no clear explanation for this discrepancy [28]. The clinical cut‐offs currently available for risk stratification, monitoring under treatment, and response assessment in patients with AL amyloidosis have all been established using the Freelite test on BNII. For example, a dFLC ≥ 50 mg/L has been defined for monitoring newly‐diagnosed AL patients [29], although recent studies have defined response criteria in patients with lower dFLC [30, 31]. It is likely that generalization of clinical cut‐offs for use outside of the system in which they were established results in erroneous clinical interpretation [32, 33, 34]. Indeed, N‐Latex values are generally lower than Freelite, and ELISA values are even lower, but both show significantly higher reproducibility [17, 20, 26, 35, 36]. It is well known that FLC concentrations measured by turbidimetry/nephelometry are higher than those measured by ELISA, probably due to FLC polymerization [20, 36]. In our study, dFLC measured by ELISA were 64% to 76% lower than N‐Latex values (median decrease for κ and λ AL respectively). However, ELISA values have been demonstrated to perfectly agree with measurable mFLC peak on sEP [20]. This suggests an overestimation of iFLC concentration by nephelometry, which was also observed in our patient samples displaying high N‐Latex dFLC. A lower dFLC cut‐off for monitoring by ELISA should thus be defined. The expected precision of ELISA, combined with the low dFLC value distinguishing AL from control patients (11.5 mg/L, Figure 1E), may allow monitoring patients with dFLC around 25 mg/L.

In the current study, detection of a monoclonal component by N‐Latex and ELISA was found to be highly sensitive. However, our ROC analysis indicates that the sensitivity of ELISA may be improved by using a higher cut‐off for the lower reference value of the κ/λ ratio. A value of 0.56, close to the value used for CKD patients (0.46), best distinguished between λ AL patients and controls. In agreement with the study of Lutteri et al. [37], which observed a small but significant increase of the κ/λ ratio from CKD stage 1, this may be explained by renal dysfunction in patients with amyloidosis, even in those with normal or subnormal eGFR (CKD stages 1 and 2).

In line with previous reports, we observed reduced agreement of N‐Latex and ELISA for the detection and quantification of λ iFLC compared to κ iFLC [20, 36]. The discrepancy between Freelite and N‐Latex FLC concentrations was also higher for λ iFLC in the study of Palladini et al. [35]. It is thought that difficulties in measuring λ mFLC are linked to the variable immunoreactivity of monoclonal antibodies with monomers (most κ) versus dimers (most λ). Several authors have shown that detection with monoclonal antibodies (N‐Latex) is limited by epitope masking due to FLC dimerization [38, 39], as illustrated by our results in two DTT‐treated serums. Using a new approach for investigating the accuracy of FLC measurement by immunochemical methods, Caponi et al. showed that Freelite and N‐Latex differ profoundly in their capacity to detect FLC monomers and dimers, and that both methods can overestimate the level of λ mFLC [40]. These discrepancies may have important clinical implications in certain patients.

Although some individual dFLC values differed greatly between N‐Latex and ELISA, we found a good agreement during the follow‐up of five patients with AL λ amyloidosis receiving treatment. N‐Latex and ELISA did not fully agree for the time point of κ/λ ratio normalization, but we observed that these small differences would have not impacted the clinical decision. A larger study would help evaluate accurate monitoring of AL patients using ELISA.

## 5 CONCLUSION

Sebia FLC ELISA can be used for the diagnosis of patients with AL amyloidosis, but its sensitivity should be improved, probably by redefining an appropriate reference range for the κ/λ ratio. Monitoring with this assay may be possible for dFLC values below 50 mg/L. Although serum FLC quantification is a great advance for the diagnosis of FLC monoclonal gammopathies, there are caveats and cautions, in particular for oligosecretory monoclonal gammopathies, such as those underlying amyloidosis. In challenging cases, the use of multiple different methods for FLC quantification should assist in accurate management of patients.

## CONFLICT OF INTEREST

Sebia (Lisses, France) provided financial support corresponding to the cost of the FLC tests dedicated to the study and performed the ELISAs. T D has received consultant fees and research grants from The Binding Site, AKCEA, ALNYLAM, GSK, PFIZER, PROTHENA and NEURIMMUNE. V A received consulting fees from Addmedica outside of the submitted work.

## AUTHOR CONTRIBUTIONS

H A and A B F collected the samples and acquired the data. H A, A B F, V A, F L, M H D L, and T D helped in writing or reviewing the manuscript. V A, F L, F L B, K B, K E K, J D, A l M, L R, S O, M K, M B, A Z, and T D were involved in clinical diagnosis of the patients. An M and E P performed the histopathological analyses. V M F was responsible for conception, analysis, and redaction of the research.

## Data Availability

V M F had full access to all the data in the study and takes responsibility for the integrity of the data and the accuracy of the data analysis.
